# Rotational stability of modified toric intraocular lens

**DOI:** 10.1371/journal.pone.0247844

**Published:** 2021-03-01

**Authors:** Ryoko Osawa, Tetsuro Oshika, Masahiko Sano, Takuma Yuguchi, Tadayoshi Kaiya

**Affiliations:** 1 Kaiya Eye Clinic, Hamamatsu, Shizuoka, Japan; 2 Department of Ophthalmology, Faculty of Medicine, University of Tsukuba, Ibaraki, Japan; Faculty of Medicine, Cairo University, EGYPT

## Abstract

We evaluated the rotational stability of a new toric intraocular lens (IOL), HOYA XY-1 toric IOL that is an improved version of HOYA 355 toric IOL, with longer overall length (13.0 mm vs. 12.5 mm), shortened unfolding time, and texture processing of the surface of haptics. Data from 193 eyes of 165 patients (76.4 ± 8.3 years old) with preoperative corneal astigmatism exceeding 0.75 diopters who had undergone phacoemulsification and toric IOL implantation were collected and analyzed. Corneal astigmatism, refractive astigmatism, and uncorrected (UDVA) and corrected distance visual acuity (CDVA) were evaluated before and 1 day, 1 week, and 1 month after surgery. The degree of IOL decentration, IOL tilt, and toric axis misalignment was assessed at 1 day and 1 month postoperatively. Fifty eyes received AcrySof toric IOL, 51 eyes TECNIS toric IOL, 46 eyes HOYA 355 toric IOL, and 46 eyes HOYA XY-1 toric IOL. The amount of axis misalignment from the intended axis was significantly different among IOLs (p = 0.004, one-way ANOVA), and HOYA XY-1 showed significantly less amount of axis misalignment than TECNIS (p = 0.020, Tukey’s multiple comparison) and HOYA 355 (p = 0.010). The proportion of eyes that showed axis misalignment <10° at 1 month postoperatively was significantly higher with HOYA XY-1 toric IOL than with other toric IOLs (χ^2^ test, p = 0.020). HOYA XY-1 toric IOL, the modified version of HOYA 355 toric IOL, showed excellent rotational stability in comparison with other models of toric IOLs.

## Introduction

Reduction of preexisting astigmatism at the time of cataract surgery using a toric intraocular lens (IOL) has become increasingly popular and is now regarded as the integral part of modern cataract surgery by many surgeons. For the success of toric IOL implantation, placement accuracy and rotational stability are the key factors. Placement accuracy of a toric IOL during surgery has been significantly improved by the advent of digital image guidance systems [1–3]. On the other hand, postoperative rotational stability varies across different models of toric IOLs [4–7]. A previous study demonstrated that the incidence of repositioning surgery to correct misalignment of toric IOLs was significantly higher with HOYA 355 toric IOL (HOYA, Tokyo, Japan) than with AcrySof toric IOL (Alcon Laboratories, Inc., Fort Worth, TX) [6]. Another clinical study reported relatively large degree of lens rotation associated with implantation of HOYA 355 toric IOL [8].

Recently, HOYA modified its 355 toric IOL to be a new version, HOYA XY-1 toric IOL, by increasing the overall length, reducing the unfolding time, and introducing texture processing to the surface of haptics. These modifications are likely to affect the rotational stability of toric IOLs, but there has been no study to prove that hypothesis. We conducted the current study to compare the surgical outcomes of new HOYA toric IOLs with those of other models of toric IOLs.

## Patients and methods

### Patients

Data from 193 eyes of 165 patients who had been treated with phacoemulsification and implantation of a toric IOL from July 2017 to February 2020 were retrospectively collected. They had corneal astigmatism of 0.75 diopter (D) or more, and were targeted emmetropic. None of the eyes had ocular or systemic diseases that could affect the surgical outcomes including visual acuity. Eyes were excluded if there were any intraoperative complications that affect IOL stability. After surgery, the patients were followed up for at least 1 month. An informed consent in written form was obtained from each patient. The study adhered to the tenets of the Declaration of Helsinki, and the institutional review board of Kaiya Eye Clinic approved the study protocol.

### Intraocular lenses and surgery

Four models of toric IOLs were used, including AcrySof toric IOL, TECNIS toric IOL (Johnson & Johnson Vision Care, Inc., Santa Ana, CA), HOYA 355 toric IOL, and HOYA XY-1 toric IOL.

HOYA XY-1 is an improved version of HOYA 355 toric IOL, with the overall length 0.5 mm longer (13.0 mm vs. 12.5 mm), shortened unfolding time by changing the material (manufacturer’s internal data), and texture processing of the surface of haptics against smooth surface with the old model. The optic is 6.0 mm in diameter and has toricity on the posterior surface with asphericity on the anterior surface. The lens is made of hydrophobic acrylic material.

A single surgeon (TK) operated on all cases with standard phacoemulsification and IOL implantation through a 2.4-mm wound. Anterior capsulorhexis of approximately 5.0 mm in diameter was created and the IOL was implanted into the capsular bag using an injector. The VERION Image-Guided System (Alcon), which consists of a measurement module and digital marker, was employed to conduct digital marking for axis alignment of toric IOLs. Using the measurement module, a high-resolution color reference image of preoperative patient’s eye was captured, which was transferred to the digital marker. Based on multiple reference points of the conjunctiva and limbus, a digital overlay of the imported preoperative image and live-surgery image were created. The eye-tracking navigation system suppressed the influence of cyclotorsion and eye movements, and the targeted placement axis of a toric IOL is accurately projected in the right ocular of the surgeon’s microscope.

### Examinations

Preoperatively, axial length and corneal curvature were measured with IOLMaster 700 (Carl Zeiss, Germany) and IOL power was calculated using the SRK/T formula. The emmetropia was the target in all of the eyes. The IOL cylinder power and alignment axis were determined using the designated manufacturer’s online calculator programs.

Corneal astigmatism, manifest astigmatism, and uncorrected (UDVA) and corrected distance visual acuity (CDVA) were measured before and 1 day, 1 week, and 1 month after surgery. Using the swept-source anterior segment optical coherence tomography (AS-OCT, CASIA, Tomey Corp., Nagoya, Japan), IOL decentration, IOL tilt, and toric axis misalignment was measured at 1 day and 1 month postoperatively [9–11]. The toric IOL analysis tool equipped with the AS-OCT shows an image of the anterior segment with an overlapped green linear marker that can be rotated on a fulcrum automatically centered on the corneal apex [11]. The linear marker is aligned parallel to the line connecting the marking dots of the toric IOL, and the direction of this alignment is expressed in angle degrees. The corneal topography obtained from the same scan is shown together with the power of steeper and flatter meridians, their axes, as well as the amount of the resulting topographic cylinder. The rotation of the toric IOL from the intended position can be expressed by the difference in degrees between the topographic axis and the value calculated for the linear marker [11]. The examiners were blinded to the type of IOL that the eye had received.

### Statistical analysis

Numerical data are expressed as mean ± standard deviation. Statistical comparisons among multiple groups were performed using the one-way analysis of variance (ANOVA) followed by the Tukey’s multiple comparison. The categorical data were compared among groups with the χ^2^ test. Statistical analysis was conducted with SPSS Statistics for Windows software (version 26, IBM Corp., Armonk, NY, USA). A p-value of less than 0.05 was considered statistically significant.

## Results

Preoperative characteristics of patients are shown in Table 1. Fifty eyes received AcrySof toric IOL, 51 eyes TECNIS toric IOL, 46 eyes HOYA 355 toric IOL, and 46 eyes HOYA XY-1 toric IOL. No significant differences were found in the baseline characteristics of patients among the groups.

**Table 1:** Patient characteristics.

	AcrySof	TECNIS	HOYA 355	HOYA XY-1
**Eyes (n)**	50	51	46	46
**Age**	71.6 ± 10.6	77.7 ± 6.7	79.4 ± 6.0	76.0 ± 6.8
**Male/female**	32/18	32/19	21/25	28/18
**Axial length (mm)**	24.1 ± 1.4	23.5 ± 0.9	23.3 ± 0.8	23.6 ± 1.1
**Preoperative corneal cylinder (D)**	1.60 ± 0.67	1.39 ± 0.54	1.25 ± 0.41	1.31 ± 0.56
**IOL power (D)**	18.9 ± 3.7	21.0 ± 2.5	20.2 ± 2.5	20.9 ± 2.8
**Target refraction (D)**	-0.05 ± 0.23	-0.15 ± 0.22	-0.15 ± 0.24	-0.03 ± 0.18
**Type of preoperative corneal cylinder: WTR/ATR/oblique**	16/29/5	6/38/7	17/21/8	9/32/5
**Type of toric IOLs: T3/T4/T5/T6/T7**	29/10/8/2/1	40/8/3/0/0	39/7/0/0	34/7/4/1/0

Mean ± standard deviation; IOL = intraocular lens; WTR = with-the-rule astigmatism (steep corneal cylinder axis was between 60° and 120°); ATR = against-the-rule astigmatism (steep corneal cylinder axis was between 0° and 30° or 150° and 180°); oblique = oblique astigmatism (steep corneal cylinder axis was between 30° and 60° or 120° and 150°).

**Fig 1:**
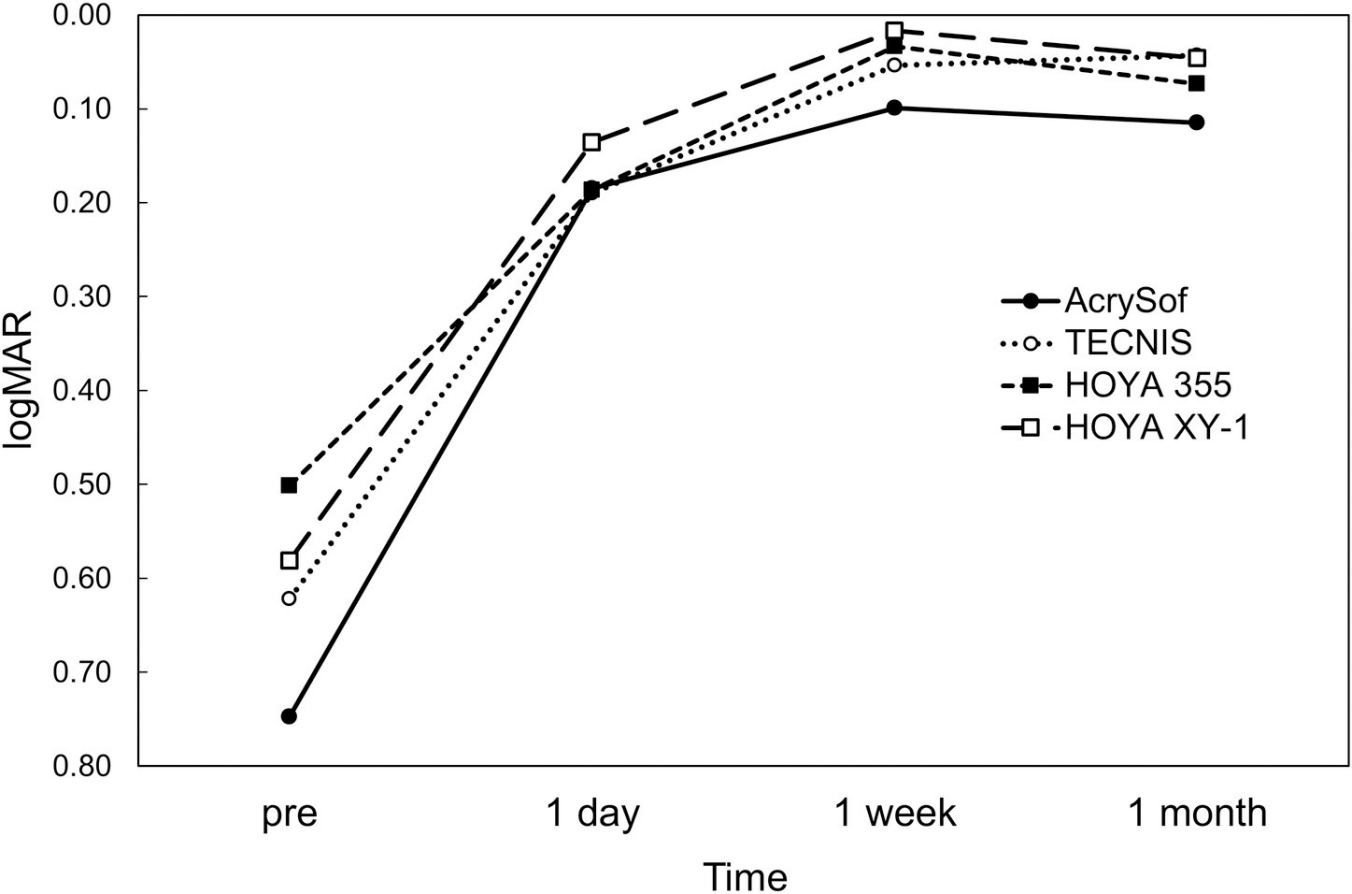
Time course of changes in uncorrected distance visual acuity.

**Fig 2:**
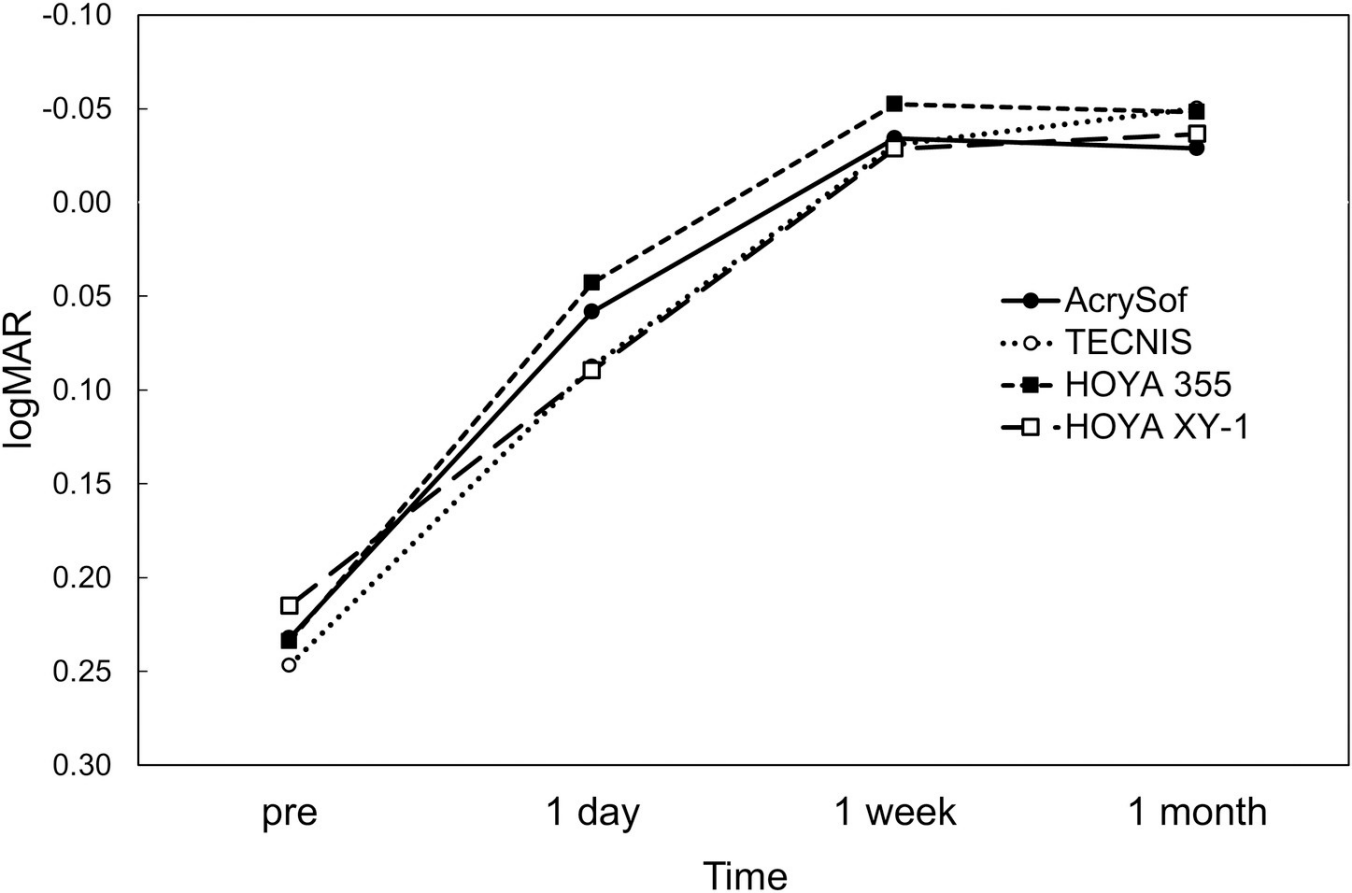
Time course of changes in corrected distance visual acuity.

**Fig 3:**
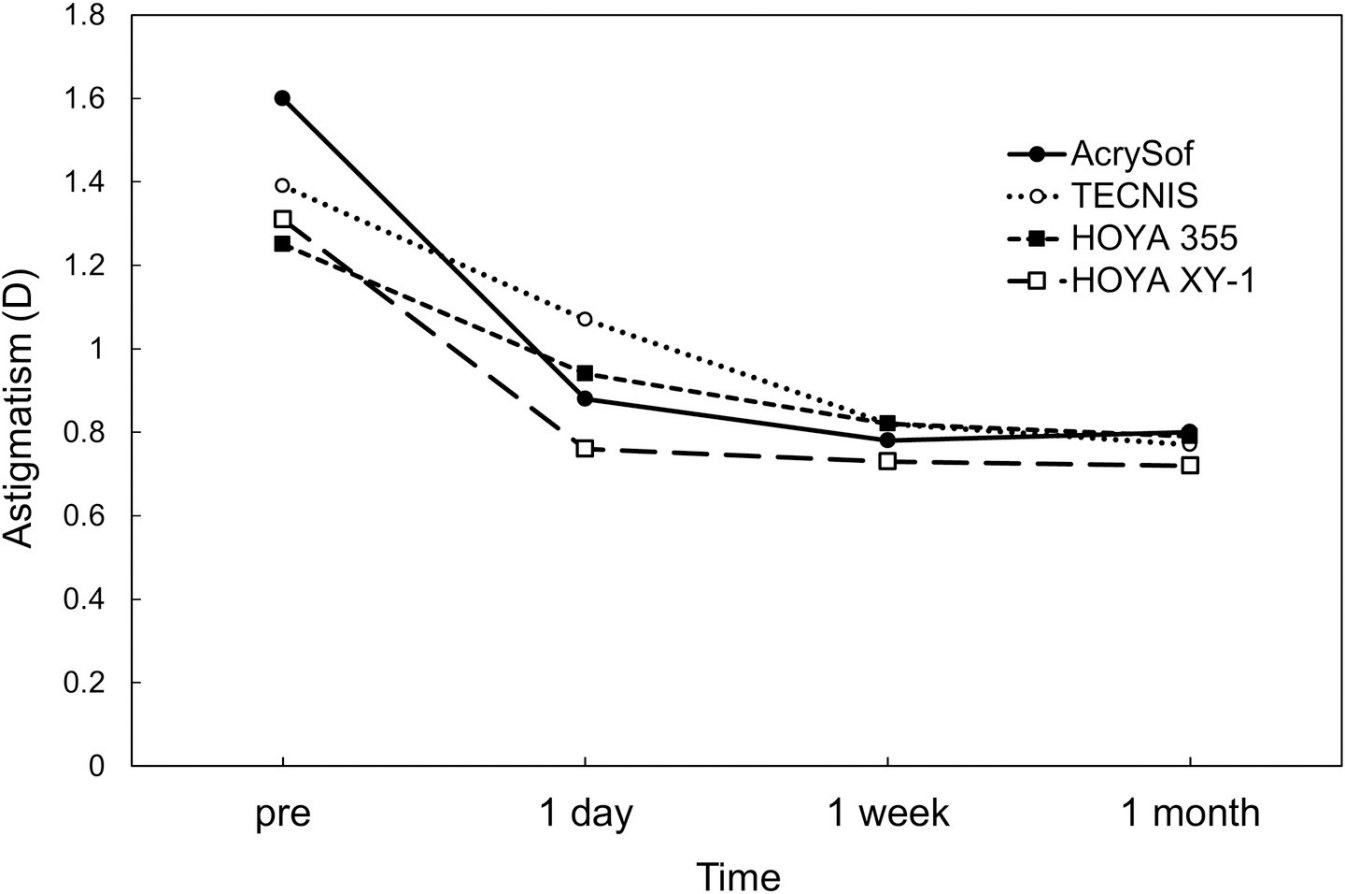
Preoperative corneal astigmatism and postoperative refractive astigmatism.

Postoperatively, there was no significant difference in UDVA (Fig 1), CDVA (Fig 2), and residual refractive astigmatism (Fig 3) among the four toric IOLs. There were no intraoperative and postoperative complications relevant to the use of toric IOLs.

**Fig 4:**
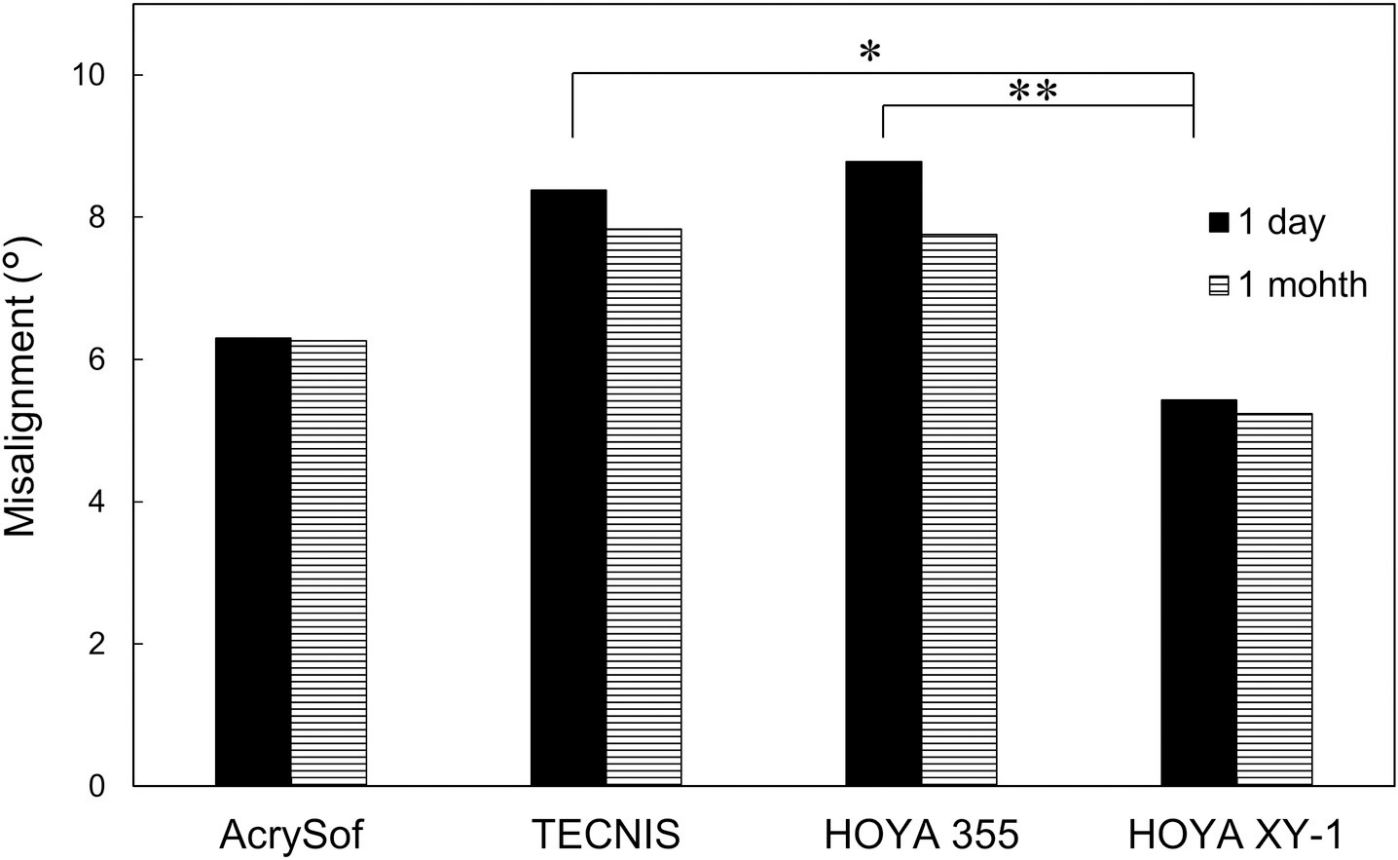
Axis misalignment from intended axis of toric intraocular lenses at 1 day and 1 month postoperatively. At day 1, there was a significant difference among IOLs (p = 0.004, one-way ANOVA), and HOYA XY-1 showed significantly less amount of axis misalignment than TECNIS (*p = 0.020, Tukey’s multiple comparison) and HOYA 355 (**p = 0.010). The difference at 1 month after surgery was marginal (p = 0.08).

**Fig 5:**
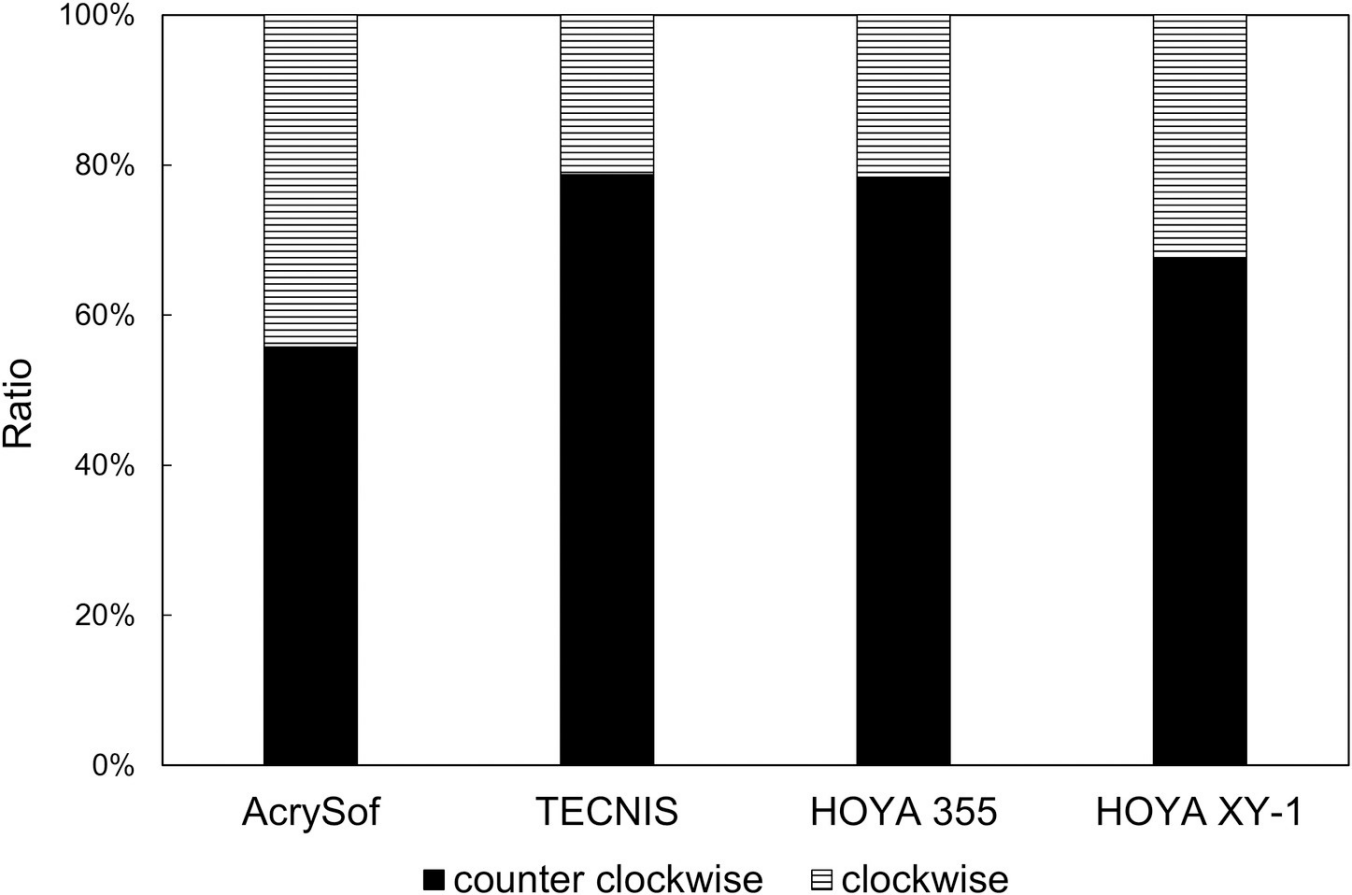
Ratio of counterclockwise and clockwise rotation of toric intraocular lenses at 1 month postoperatively.

The amount of axis misalignment from the intended axis is shown in Fig 4 and Table 2. At 1 day postoperatively, there was a significant difference among IOLs (p = 0.004, one-way ANOVA), and HOYA XY-1 showed significantly less amount of axis misalignment than TECNIS (p = 0.020, Tukey’s multiple comparison) and HOYA 355 (p = 0.010). The difference at 1 month after surgery was marginal (p = 0.08), with HOYA XY-1 showing the smallest amount of misalignment. The proportion of eyes that presented axis misalignment <10° at 1 month with AcrySof, TECNIS, HOYA 355, and HOYA XY-1 toric IOLs was 82.0% (41/50), 64.7% (33/51), 54.3% (25/46), and 89.1% (41/46), respectively; HOYA XY-1 toric IOL had significantly higher rate than other toric IOLs (χ^2^ test, p = 0.020). In all groups, more than half of eyes showed counterclockwise rotation of toric IOLs at 1 month (Fig 5). The ratio did not differ significantly among groups (p = 0.456). The repositioning surgery to correct axis misalignment was not carried out in all groups.

**Table 2:** Misalignment, tilt, and decentration of toric intraocular lenses.

	AcrySof	TECNIS	HOYA 355	HOYA XY-1
**Misalignment (°)**				
**1 day**	6.30 ± 4.80	8.38 ± 6.84	8.78 ± 6.54	5.43 ± 4.67
**1 month**	6.26 ± 4.77	7.83 ± 7.07	7.76 ± 5.67	5.24 ± 4.58
**Tilt (°)**				
**1 day**	4.60 ± 1.64	4.84 ± 1.17	5.12 ± 1.24	4.79 ± 1.28
**1 month**	4.81 ± 1.49	5.18 ± 1.21	5.12 ± 1.24	4.80 ± 1.11
**Decentration (mm)**				
**1 day**	0.21 ± 0.11	0.18 ± 0.12	0.18 ± 0.11	0.16 ±0.09
**1 month**	0.30 ± 0.60	0.16 ± 0.10	0.21 ± 0.15	0.18 ± 0.11

Mean ± standard deviation.

There was no statistically significant correlation between axial length and the among of misalignment at 1 month in all cases (p = 0.977). The correlation was not significant in individual IOL groups (p = 0.377 for AcrySof, p = 0.366 for TECNIS, p = 0.731 for HOYA 355, and p = 0.313 for HOYA XY-1).

The degree of IOL tilt and decentration at 1 day and 1 month postoperatively is summarized in Table 2. There was no significant difference among groups in tilt (1 day p = 0.305, 1 month p = 0.390) and decentration (1 day p = 0.530, 1 month p = 0.164).

## Discussion

HOYA XY-1 toric IOL is the improved version of HOYA 355 toric IOL. The modifications include longer overall length (13.0 mm versus 12.5 mm), faster unfolding once released in the eye, and texture processing of the surface of haptics against smooth surface with the old model. The current study demonstrated that these modifications resulted in significantly better rotational stability after surgery. The amount of misalignment at 1 day and 1 month postoperatively was reduced from 8.78 ± 6.54° (HOYA 355) to 5.43 ± 4.67° (HOYA XY-1) and 7.76 ± 5.67° (355) to 5.24 ± 4.58° (XY-1), respectively. The proportion of eyes that showed axis misalignment <10° was significantly higher with HOYA XY-1 toric IOL than other toric IOLs, including TECNIS and HOYA 355 toric IOLs. At present, we have no data regarding which of the above three refinements played the most important role in improving rotational stability of HOYA XY-1 toric IOL. A previous study indicated that the greatest axis misalignment of a toric IOL occurred as an IOL rotation from the end of surgery to one hour after surgery, and that IOL orientation remained very stable thereafter [12]. Theoretically, each of those three improvements (increased overall length, faster unfolding, and texture finishing) can contribute to the better rotational stability of a toric IOL immediately after surgery.

The proportion of eyes that presented axis misalignment <10° at 1 month with AcrySof, TECNIS, HOYA 355, and HOYA XY-1 toric IOLs was 82.0% (41/50), 64.7% (33/51), 54.3% (25/46), and 89.1% (41/46), respectively. The TECNIS and HOYA 355 toric IOLs showed significantly lower rate than AcrySof toric IOL. This result is in good accordance with those of previous studies. A retrospective comparative study found that the rate of repositioning surgery was significantly higher with TECNIS and HOYA 355 toric IOLs than AcrySof toric IOL [6]. It was also reported that AcrySof toric IOL was less likely to rotate postoperatively than TECNIS toric IOL [5], and that TECNIS platform toric IOL was more likely to rotate and to require surgical repositioning than the Alcon Restor toric IOL [7].

Regarding the direction of axis misalignment of toric IOLs, a previous study reported that AcrySof toric IOLs presented clockwise rotation in 76.2% and anti-clockwise rotation in 23.8% [13]. Another study, on the other hand, found no trend for either clockwise or counterclockwise rotation with AcrySof toric IOLs [14]. By analyzing the on-line database, it was shown that TECNIS toric IOL was more likely to be rotated in a counterclockwise direction, but no such bias was found with AcrySof toric IOL [4]. In the present study, anti-clockwise misdirection was more frequent than clockwise misdirection with all four toric IOLs, but no significant differences were observed in the incidence between groups.

In this study, the amount of IOL tilt and decentration of four toric IOLs was approximately 5° and 0.2 mm, respectively, and there were no significant differences among IOLs. The influence of IOL decentration and tilt on visual function varies depending on the design of IOLs [15]. For toric IOLs, decentration and tilt can result in less predictable correction of astigmatism [16]. An experimental study using a ray tracing model demonstrated that the alignment and toricity as well as orientation of tilt caused over-correction or under-correction of astigmatism by toric IOLs [16]. A review analysis of studies about IOL decentration and tilt demonstrated that 2–3° tilt and 0.2–0.3 mm decentration are common and practically unnoticed for IOLs with any design [17]. A model eye investigation indicated that more than 5° tilt and more than 1 mm IOL decentration can be visually significant, inducing oblique astigmatism [18]. According to optical simulations with consideration of various visual aspects such as corneal aberrations and pupil function, decentration of 0.5 mm or greater could be a source of considerable visual symptoms [19]. The tilt of a 28 D aspheric IOL by 5° and 10° was calculated to induce astigmatism of 0.14 D and 0.56 D, respectively [16]. In the literature, an average of approximately 5° tilt and 0.2 mm decentration of IOLs implanted in the capsular bag have been reported [20–24]. Judging from these previous studies, the degree of tilt and decentration found with the toric IOLs used in our study is within an acceptable range, and its impacts on postoperative visual functions seem minimal.

There are several limitations to this study. First, because this was a retrospective study, random assignment of eyes to one of four toric IOLs was not conducted. Second, the part of study using HOYA 355 and XY-1 IOLs was not parallel, but sequential. This was because HOYA 355 toric IOL was pulled out of the market on the launch of HOYA XY-1 toric IOL, making the use of both IOLs at the same time impossible. Third, the follow-up period of patients was 1 month, and longer clinical outcomes were not assessed. The orientation of toric IOLs, however, is highly stable after 1 day postoperatively [12], and thus we suppose that results at 1 month postoperatively are able to extrapolate the longer-term rotational stability of toric IOLs. Third, the amount of postoperative misalignment found in the current study was somewhat greater than those previously reported [25]. The degree of misalignment, however, does vary significantly among studies, indicating that simple comparison of data between different studies with various background conditions of patients and surgical techniques would be difficult [26].

## Conclusions

We retrospectively assessed the surgical outcomes of four toric IOLs. HOYA XY-1 toric IOL, the improved version of HOYA 355, showed excellent rotational stability in comparison with other models of toric IOLs.

## References

[1] Abdel Hamid Elhofi Hany Ahmed Helaly. Comparison between digital and manual marking for toric intraocular lenses: A randomized trial. Medicine (Baltimore). 2015;94: e1618. 10.1097/MD.0000000000001618 26402830 PMC4635770

[2] Titiyal JS, Kaur M, Jose CP, Falera R, Kinkar A, Bageshwar LM. Comparative evaluation of toric intraocular lens alignment and visual quality with image-guided surgery and conventional three-step manual marking. Clin Ophthalmol. 2018;12: 747–753. 10.2147/OPTH.S164175 29731603 PMC5923224

[3] Panagiotopoulou EK, Ntonti P, Gkika M, Konstantinidis A, Perente I, Dardabounis D, et al. Image-guided lens extraction surgery: a systematic review. Int J Ophthalmol. 2019;12: 135–151. 10.18240/ijo.2019.01.21 30662853 PMC6326930

[4] Potvin R, Kramer BA, Hardten DR, Berdahl JP. Toric intraocular lens orientation and residual refractive astigmatism: an analysis. Clin Ophthalmol. 2016;10: 1829–1836. 10.2147/OPTH.S114118 27703323 PMC5036610

[5] Lee BS, Chang DF. Comparison of the rotational stability of two toric intraocular lenses in 1273 consecutive eyes. Ophthalmology. 2018;125: 1325–1331. 10.1016/j.ophtha.2018.02.012 29544960

[6] Oshika T, Fujita Y, Hirota A, Inamura M, Inoue Y, Miyata K, et al. Comparison of incidence of repositioning surgery to correct misalignment with three toric intraocular lenses. Eur J Ophthalmol. 2020;30: 680–684. 10.1177/1120672119834469 30841757

[7] Lee BS, Onishi AC, Chang DF. Comparison of rotational stability and repositioning rates of two presbyopia-correcting and two monofocal toric intraocular lenses. J Cataract Refract Surg. 2020 11 19. 10.1097/j.jcrs.0000000000000497 Epub ahead of print. . 33181626

[8] Bissen-Miyajima H, Negishi K, Hieda O, Kinoshita S. Microincision hydrophobic acrylic aspheric toric intraocular lens for astigmatism and cataract correction. J Refract Surg. 2015;31: 358–364. 10.3928/1081597X-20150521-01 26046701

[9] Kimura S, Morizane Y, Shiode Y, Hirano M, Doi S, Toshima S, et al. Assessment of tilt and decentration of crystalline lens and intraocular lens relative to the corneal topographic axis using anterior segment optical coherence tomography. PLoS ONE. 2017;12: e0184066. 10.1371/journal.pone.0184066 28863141 PMC5581187

[10] Sato T, Shibata S, Yoshida M, Hayashi K. Short-term dynamics after single- and three-piece acrylic intraocular lens implantation: a swept-source anterior segment optical coherence tomography study. Sci Rep. 2018;8: 10230. 10.1038/s41598-018-28609-1 29980770 PMC6035277

[11] Lucisano A, Ferrise M, Balestrieri M, Busin M, Scorcia V. Evaluation of postoperative toric intraocular lens alignment with anterior segment optical coherence tomography. J Cataract Refract Surg. 2017;43: 1007–1009. 10.1016/j.jcrs.2017.05.025 28917397

[12] Inoue Y, Takehara H, Oshika T. Axis misalignment of toric intraocular lens: Placement error and postoperative rotation. Ophthalmology. 2017;124: 1424–1425. 10.1016/j.ophtha.2017.05.025 28647201

[13] Shah GD, Praveen MR, Vasavada AR, Vasavada VA, Rampal G, Shastry LR. Rotational stability of a toric intraocular lens: influence of axial length and alignment in the capsular bag. J Cataract Refract Surg. 2012;38: 54–59. 10.1016/j.jcrs.2011.08.028 22055077

[14] Zuberbuhler B, Signer T, Gale R, Haefliger E. Rotational stability of the AcrySof SA60TT toric intraocular lenses: a cohort study. BMC Ophthalmol. 2008;8: 8. 10.1186/1471-2415-8-8 18460196 PMC2408563

[15] Ashena Z, Maqsood S, Ahmed SN, Nanavaty MA. Effect of intraocular lens tilt and decentration on visual acuity, dysphotopsia and wavefront aberrations. Vision (Basel). 2020;4: 41. 10.3390/vision4030041 32937750 PMC7559075

[16] Weikert MP, Golla A, Wang L. Astigmatism induced by intraocular lens tilt evaluated via ray tracing. J Cataract Refract Surg. 2018;44: 745–749. 10.1016/j.jcrs.2018.04.035 29861054

[17] Ale J.B. Intraocular lens tilt and decentration: A concern for contemporary IOL designs. Nepal J Ophthalmol. 2011;3: 68–77. 10.3126/nepjoph.v3i1.4281 21505548

[18] Korynta J, Bok J, Cendelin J, Michalova K. Computer modeling of visual impairment caused by intraocular lens misalignment. J. Cataract Refract Surg. 1999;25: 100–105. 10.1016/s0886-3350(99)80019-4 9888085

[19] Lawu T, Mukai K, Matsushima H, Senoo T. Effects of decentration and tilt on the optical performance of 6 aspheric intraocular lens designs in a model eye. J. Cataract Refract Surg. 2019;45: 662–668. 10.1016/j.jcrs.2018.10.049 30876781

[20] Kimura S, Morizane Y, Shiode Y, Hirano M, Doi S, Toshima S, et al. Assessment of tilt and decentration of crystalline lens and intraocular lens relative to the corneal topographic axis using anterior segment optical coherence tomography. PLoS ONE. 2017;12: e0184066. 10.1371/journal.pone.0184066 28863141 PMC5581187

[21] Sato T. Shibata S, Yoshida M, Hayashi K. Short-term dynamics after single- and three-piece acrylic intraocular lens implantation: A swept-source anterior segment optical coherence tomography study. Sci Rep. 2018;8: 10230. 10.1038/s41598-018-28609-1 29980770 PMC6035277

[22] Hirnschall N, Buehren T, Bajramovic F, Trost M, Teuber T, Findl O. Prediction of postoperative intraocular lens tilt using swept-source optical coherence tomography. J Cataract Refract Surg. 2017;43: 732–736. 10.1016/j.jcrs.2017.01.026 28732605

[23] Wang L, Guimaraes de Souza R, Weikert MP, Koch DD. Evaluation of crystalline lens and intraocular lens tilt using a swept-source optical coherence tomography biometer. J Cataract Refract Surg. 2019;45: 35–40. 10.1016/j.jcrs.2018.08.025 30309775

[24] Chen X, Gu X, Wang W, Xiao W, Jin G, Wang L, et al. Characteristics and factors associated with intraocular lens tilt and decentration after cataract surgery. J Cataract Refract Surg. 2020;46:1126–1131. 10.1097/j.jcrs.0000000000000219 32352251

[25] Zhou F, Jiang W, Lin Z, Li X, Li J, Lin H, et al. Comparative meta-analysis of toric intraocular lens alignment accuracy in cataract patients: Image-guided system versus manual marking. J Cataract Refract Surg. 2019;45: 1340–1345. 10.1016/j.jcrs.2019.03.030 31470944

[26] Visser N, Bauer NJ, Nuijts RM. Toric intraocular lenses: historical overview, patient selection, IOL calculation, surgical techniques, clinical outcomes, and complications. J Cataract Refract Surg. 2013;39: 624–37. 10.1016/j.jcrs.2013.02.020 23522584

